# The Value of Intraoperative Near-Infrared Fluorescence Imaging Based on Enhanced Permeability and Retention of Indocyanine Green: Feasibility and False-Positives in Ovarian Cancer

**DOI:** 10.1371/journal.pone.0129766

**Published:** 2015-06-25

**Authors:** Quirijn R. J. G. Tummers, Charlotte E. S. Hoogstins, Alexander A. W. Peters, Cor D. de Kroon, J. Baptist M. Z. Trimbos, Cornelis J. H. van de Velde, John V. Frangioni, Alexander L. Vahrmeijer, Katja N. Gaarenstroom

**Affiliations:** 1 Department of Surgery, Leiden University Medical Center, Leiden, The Netherlands; 2 Department of Gynecology, Leiden University Medical Center, Leiden, The Netherlands; 3 Department of Medicine, Division of Hematology/Oncology, Beth Israel Deaconess Medical Center, Boston, Massachusetts, United States of America; 4 Department of Radiology, Beth Israel Deaconess Medical Center, Boston, Massachusetts, United States of America; 5 Curadel, LLC, Worcester, Massachusetts, United States of America; Fondazione IRCCS Istituto Nazionale dei Tumori, ITALY

## Abstract

**Objective:**

In ovarian cancer, two of the most important prognostic factors for survival are completeness of staging and completeness of cytoreductive surgery. Therefore, intra-operative visualization of tumor lesions is of great importance. Preclinical data already demonstrated tumor visualization in a mouse-model using near-infrared (NIR) fluorescence imaging and indocyanine green (ICG) as a result of enhanced permeability and retention (EPR). The aim of this study was to determine feasibility of intraoperative ovarian cancer metastases imaging using NIR fluorescence imaging and ICG in a clinical setting.

**Methods:**

Ten patients suspected of ovarian cancer scheduled for staging or cytoreductive surgery were included. Patients received 20 mg ICG intravenously after opening the abdominal cavity. The mini-FLARE NIR fluorescence imaging system was used to detect NIR fluorescent lesions.

**Results:**

6 out of 10 patients had malignant disease of the ovary or fallopian tube, of which 2 had metastatic disease outside the pelvis. Eight metastatic lesions were detected in these 2 patients, which were all NIR fluorescent. However, 13 non-malignant lesions were also NIR fluorescent, resulting in a false-positive rate of 62%. There was no significant difference in tumor-to-background ratio between malignant and benign lesions (2.0 vs 2.0; P=0.99).

**Conclusions:**

This is the first clinical trial demonstrating intraoperative detection of ovarian cancer metastases using NIR fluorescence imaging and ICG. Despite detection of all malignant lesions, a high false-positive rate was observed. Therefore, NIR fluorescence imaging using ICG based on the EPR effect is not satisfactory for the detection of ovarian cancer metastases. The need for tumor-specific intraoperative agents remains.

**Trial Registration:**

ISRCTN Registry ISRCTN16945066

## Introduction

Ovarian cancer has a worldwide incidence of 225,500 making it the 6th most common cancer in women. With 140,200 deaths worldwide per year, it has the highest mortality rates amongst all gynaecological cancers [1–3]. In general, ovarian cancer can be classified as early stage (FIGO I to IIa) or advanced stage (FIGO IIb to IV). Prognosis and treatment are mainly dependent on this classification.

Early stage ovarian cancer has a 5 year survival of 75–100%, with the most important factors influencing survival being differentiation grade of the tumor and the completeness of staging [1]. During surgical staging, blind biopsy samples of predefined areas and biopsy samples of suspected lesions are obtained. The primary aim of the staging procedure is to determine whether there is occult metastatic disease not primarily visible by the naked eye. When no metastases are present, resection of the primary tumor is the treatment of choice and chemotherapy can be avoided [4]. When metastases are present, surgical resection is supplemented with chemotherapy. Optimal staging has been shown to improve survival in low stage ovarian cancer because it discriminates true early stage ovarian cancer from occult tumor spread, which results in more advanced disease [4].

However, most patients (around 75%) present with advanced disease [5]. The most important prognostic factor for overall survival in advanced stage disease is the amount of residual tumor after cytoreductive surgery [6–8]. Therefore complete cytoreduction, defined as no visible residual tumor left after surgery, or optimal cytoreduction, not consistently defined as a maximal diameter of residual tumor of 0–2 cm [5,9–11], is the goal for advanced stage ovarian cancer surgery.

In order to achieve either optimal staging or complete or optimal cytoreduction, visualization of tumor lesions is of great importance. With imaging modalities such as CT and MRI, pre-operative identification and localization of tumor lesions is reasonably achievable, however intraoperative visualization of tumor tissue can be challenging.

Near-infrared (NIR) fluorescence imaging is a promising technique to assist in the real time intraoperative identification of malignant lesions [12]. This technique makes use of NIR fluorescent light (700–900 nm) emitted by contrast agents after excitation by an imaging system able to detect this NIR fluorescent signal. NIR fluorescence is relatively easy and provides sufficient contrast due to high tissue penetration and low auto-fluorescence [13].

For tumor identification, it is essential that contrast agents accumulate in tumor tissue either actively or passively. Active accumulation can be achieved by targeting ligands that are over-expressed on tumor tissue. Van Dam et al. were the first to show tumor identification using a folate receptor alpha (FR-a) targeting agent [14]. In their series they were able to identify tumor tissue in 3 patients with FR-a positive ovarian cancer intraoperatively. However, these results have not yet been reproduced in other studies using a FR-a or different targeting agents. A possible cause for this may be the expensive and time-consuming nature of the development of these tumor-specific agents. Therefore, it is of great importance to exploit clinically available contrast agents, such as indocyanine green (ICG) [15].

In vivo ICG binds to serum proteins and therefore behaves as a macromolecule in the circulation. It is known that macromolecules accumulate in tumor tissue due to increased vascular permeability and reduced drainage. This phenomenon is called the "enhanced permeability and retention" (EPR) effect and has been observed in most solid tumors [16,17].

Clinical feasibility trials using this effect with ICG in breast cancer patients in a pre-operative diagnostic setting and in gastric cancer patients during endoscopic surgery showed that it was possible to distinguish tumor from surrounding tissue [18–23]. In addition, Kosaka et al.[24] detected small ovarian (1–2 mm in size) cancer implants using NIR fluorescent imaging after intravenous (IV) administration of ICG in a mouse model. Pathophysiological heterogeneity of solid tumors, for examples in size, presence of necrosis, or presence of vascular mediators may influence accumulation of macromolecules in tumor tissue [25,26]. It is therefore not clear if all preclinical results can be translated to the clinic.

The primairy aim of the current study was to determine the feasibility of ovarian cancer metastases detection using ICG and NIR fluorescence imaging in a clinical setting. Secondary aim was to assess concordance between fluorescence signal and tumor status on histopathology. In addition, we sought to determine if a sufficient tumor-to-background ratio (TBR), based on the EPR effect, could be obtained to discriminate between malignant and non-malignant tissue.

## Material and Methods

### Tracer preparation

ICG was prepared in the operating room following preparation instructions of the institutional pharmacist. ICG (25 mg vials, purchased from Pulsion Medical Systems Munich, Germany) was diluted in 10 cc of sterile water for injection to yield a 2.5mg/ml (3.2 mM) stock solution.

### Clinical trial

The study protocol (S1 and S2 Protocols) was approved by the Local Medical Ethics Committee of the Leiden University Medical Center (LUMC) on 27-06-2012 and was performed in accordance with the ethical standards of the Helsinki Declaration of 1975. Due to administrational error, trail registration was performed after the start of the study (date trial registration 11-09-2014; ISRCTN16945066). This non-randomised study adheres to the Transparent Reporting of Evaluations with Non-Randomised designs (TREND) guidelines (S1 TREND Checklist). The authors confirm that all ongoing and related trials for this intervention are registered. Because this study was set up as feasibility study, no formal sample size could be calculated. Determination of feasibility was expected after inclusion of 15 patients. In case apparent non-feasibility was observed, earlier termination of the study could be performed.

Eventually, ten patients presenting at the department of Gynaecological Oncology of LUMC between 14 October 2012 and 11 December 2013 suspected of either early stage ovarian cancer scheduled to undergo staging surgery or of advanced ovarian cancer scheduled to undergo cytoreductive surgery, were included. All surgical procedures were performed by laparotomy through a midline abdominal incision. All patients gave written informed consent. Exclusion criteria were pregnancy, severe renal insufficiency (GFR < 55 mL/min/1.73 m^2^), or an allergy to iodine or ICG.

In the operating theatre, after opening of the abdominal cavity, 20 mg of ICG was administered intravenously as single bolus by the anaesthesiologist. The average time between administration of ICG and imaging of the first lesion was 37 minutes. The last lesion was imaged on average 141 minutes post-administration of ICG. First the surgical field was searched for metastases visible by the naked eye. After resection of the primary tumor, uterus and ovaries, the Mini-FLARE was used to identify NIR fluorescent signals. When a fluorescent signal was observed, the operating surgeon performed a biopsy or resection of the fluorescent tissue, unless this would jeopardize patient health or success of surgery. In case of non-fluorescence, only macroscopically suspected lesions were resected. Resected specimens were marked as fluorescent or non-fluorescent and were routinely examined by a pathologist for the presence of malignant cells.

### Intraoperative Near-Infrared Fluorescence Imaging

Intraoperative imaging procedures were performed using the Mini-Fluorescence-Assisted Resection and Exploration (Mini-FLARE) image-guided surgery system, as described earlier [27]. Briefly, the system consists of 2 wavelength isolated light sources: a “white” light source, generating 26,600 lx of 400 to 650 nm light, and a “near-infrared” light source, generating 1.08 mW/came of ≈ 760 nm light. Color video and NIR fluorescence images are simultaneously acquired and displayed in real time using custom optics and software that separate the color video and NIR fluorescence images. A pseudo-colored (lime green) merged image of the color video and NIR fluorescence images is also displayed. The imaging head is attached to a flexible gooseneck arm, which permits positioning of the imaging head at extreme angles virtually anywhere over the surgical field. For intraoperative use, the imaging head and imaging system pole stand are wrapped in a sterile shield and drape (Medical Technique Inc., Tucson, AZ).

### Statistical Analysis

For statistical analysis, SPSS statistical software package (Version 20.0, Chicago, IL) was used. TBRs were calculated by dividing the fluorescent signal of the tumor by fluorescent signal of surrounding tissue. Patient age was reported in median, standard deviation (SD), and range, and TBR was reported in mean, SD, and range. To compare patient characteristics, independent samples t-test and chi-square tests were used. To compare TBR and background signal between malignant and benign lesions, independent samples t-test was used. P < 0.05 was considered significant.

## Results

### Patient characteristics

**Fig 1:**
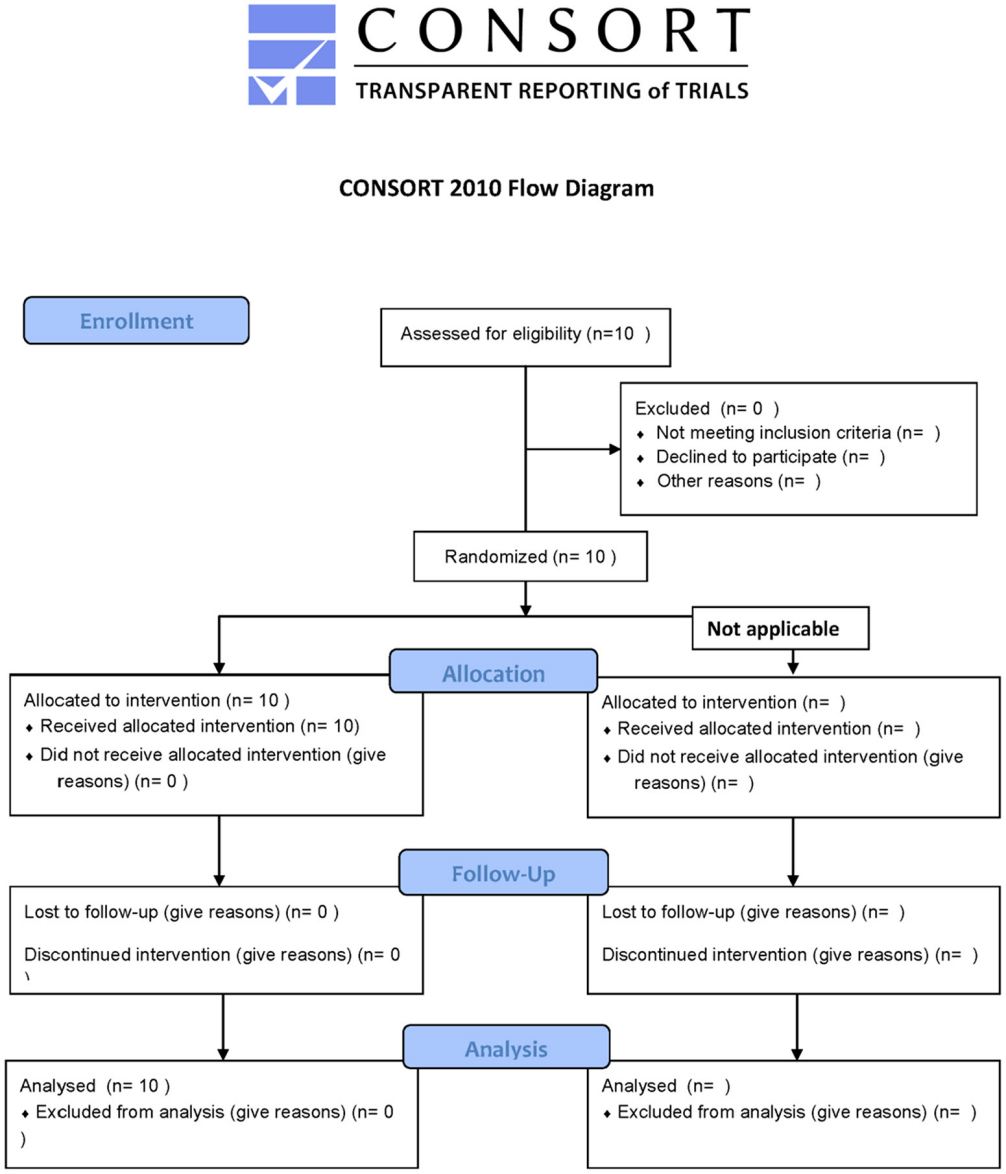
CONSORT flow diagram for patient enrollment.

Ten patients were included in this study. Fig 1 shows the CONSORT flow diagram for enrollment of patients. Median age was 58 years (range 42–74). Table 1 shows the patient characteristics. Seven patients underwent a staging procedure (70%), and 3 patients underwent a debulking procedure (30%) All staging and debulking procedures were open procedures.

**Table 1:** Patient and tumor characteristics.

Study number	Age	Origin	Histologic type	FIGO stage	Surgical procedure	Metastases found during procedure
1	58	Ovary	Clearcell	1a	Staging	No
2	69	*Benign disease*	n.a.	n.a.	Staging	No
3	74	*Benign disease*	n.a.	n.a.	Staging	No
4	73	Ovary	Serous	3c	Cytoreduction	Yes
5	42	Ovary	Serous	2c	Cytoreduction	Yes
6	50	Endometrium	Endometrioid	3a	Staging	No*
7	73	*Benign disease*	n.a.	n.a.	Staging	No
8	58	Ovary	Endometrioid	1a	Staging	No
9	54	Fallopian Tube	Serous	1a	Staging	No
10	50	Ovary	Serous, Mucinous	2c	Cytoreduction	No†

* Staging was performed because an ovarian metastasis was detected at pathology after an earlier polipectomy procedure

^†^ No biopsies were taken due to the presence of adhesions and tumor spill during the procedure, therefore defining the tumor stage as IIc with a concomitant indication for postoperative chemotherapy

Histological assessment by the pathologist of the resected lesions confirmed the following diagnosis: 6 patients were diagnosed with either ovarian cancer (5) or cancer of the fallopian tube (1), of which the following subtypes were diagnosed: serous (3), clear-cell (1), endometrioid (1), mixed (1); one patient was diagnosed with endometrial cancer (endometriod type); and 3 patients had benign ovarian tumors. An overview of the final histological diagnoses and FIGO stage is given in Table 1.

### Metastatic lesions

2 out of the 6 patients with malignant disease of the ovary or fallopian tube, suffered from histologically proven metastatic disease (patients #4 and #5). A total of 8 metastatic lesions, confirmed by the pathologist, were found in these 2 patients (4 lesions in both #4 and #5). Lesions were localized at the pouch of Douglas (N = 3), bladder peritoneum (N = 2), para iliacal lymphnodes (N = 2) and omentum (N = 1).

### NIR Fluorescence imaging

**Fig 2:**
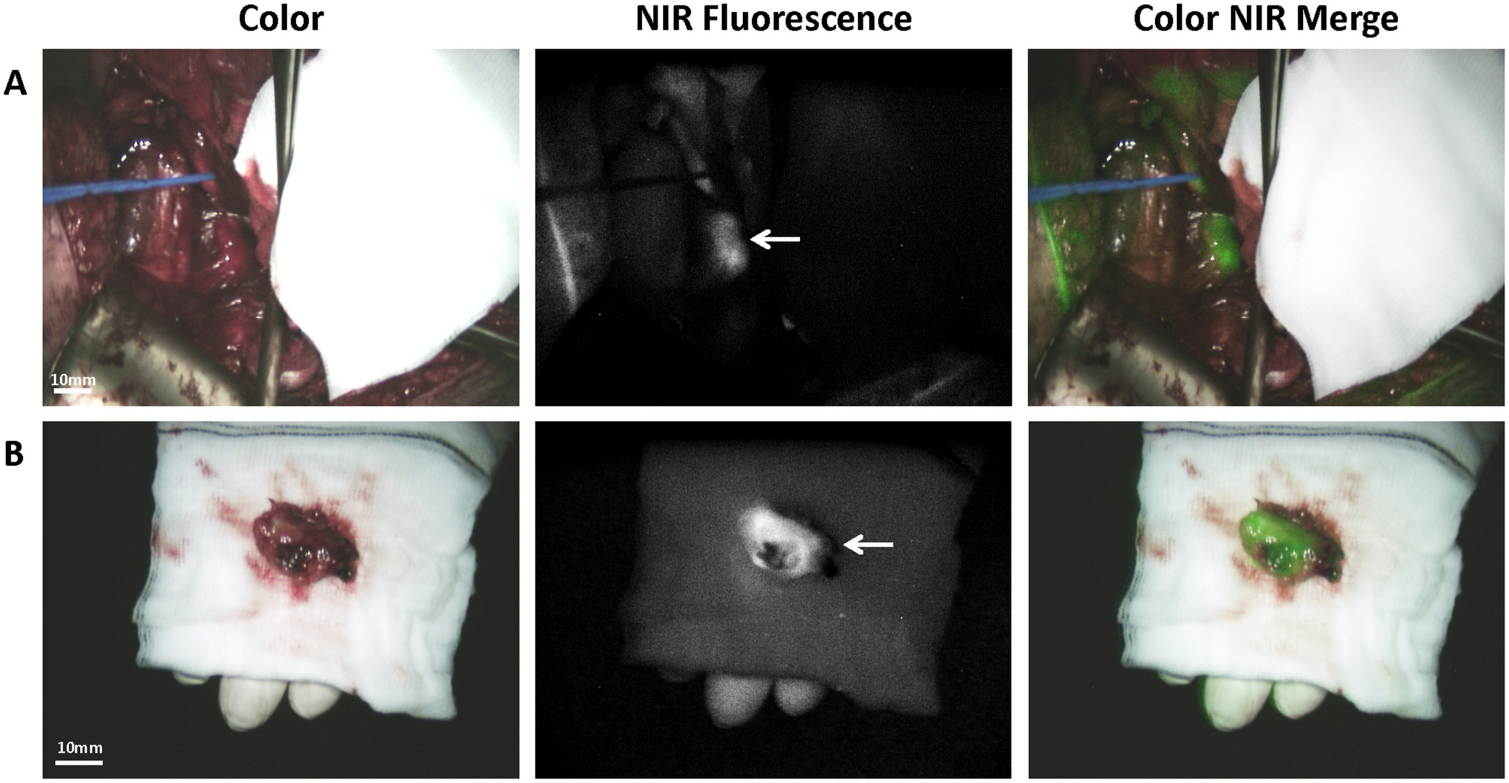
Identification of ovarian cancer metastases using NIR fluorescence imaging. A. Identification of ovarian cancer metastases located in a lymph node next to the right iliac vein (arrow) using NIR fluorescence imaging. The lesion was found histologically to be a metastasis of serous adenocarcinoma. B. *Ex vivo* imaging of the same ovarian cancer metastases located in a lymph node next to the right iliac vein (arrow).

**Fig 3:**
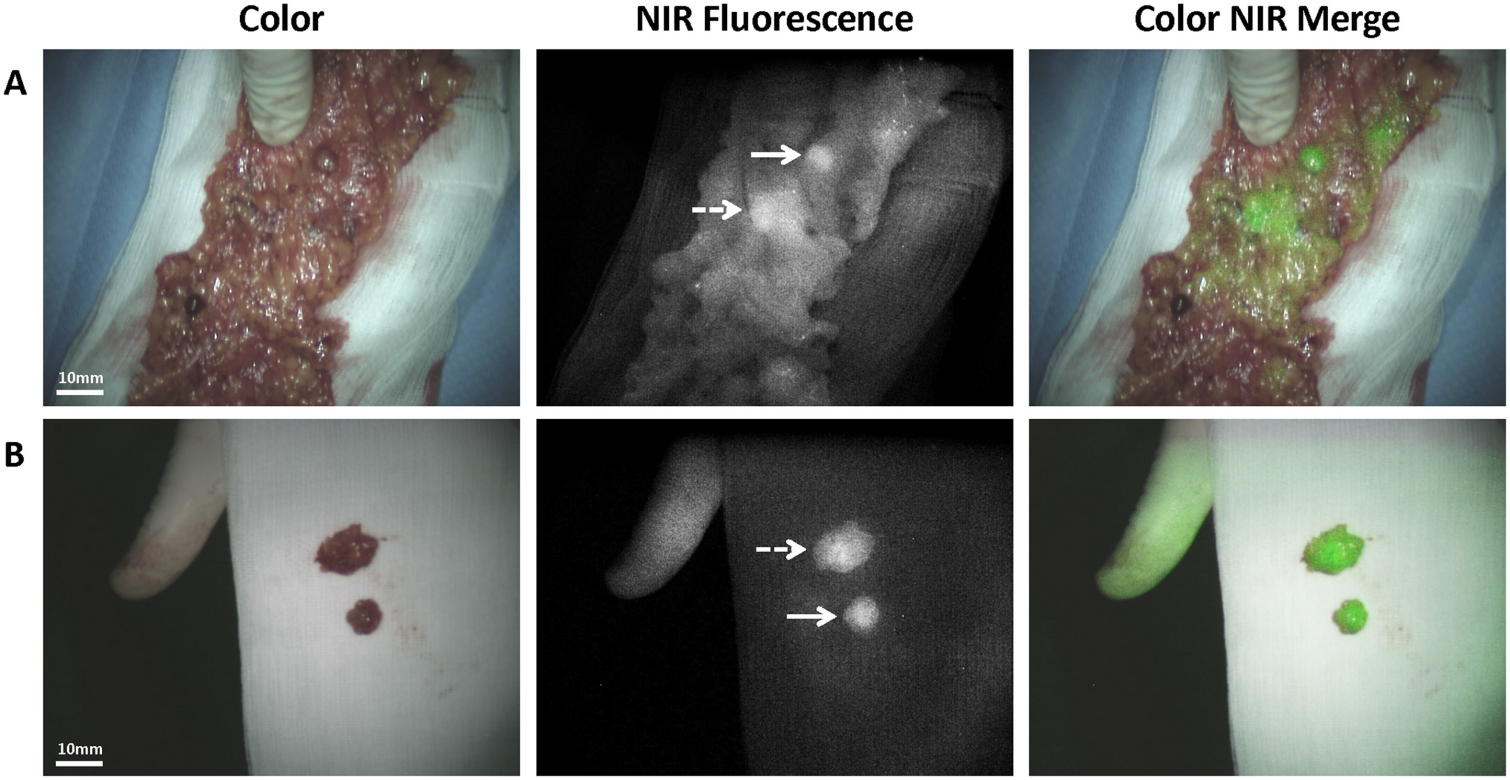
Identification of ovarian cancer omental metastases using NIR fluorescence imaging. A. Identification of 2 ovarian cancer metastases located in the greater omentum (arrow and dashed arrow) using NIR fluorescence imaging. B. Imaging of the same two NIR fluorescent lesions removed from the omentum (arrow and dashed arrow). Both lesions were found histologically to be metastases of serous adenocarcinoma.

A total of 21 fluorescent lesions were identified. Fig 2A shows an example of a clinically suspected lesion, which was NIR fluorescent. This lesion was anatomically located next to the right iliac vein. Fig 2B shows the *ex vivo* images of the same NIR fluorescent lesion. This lesion was found histologically to be a metastasis of serous adenocarcinoma of the ovary. Fig 3A and 3B show 2 NIR fluorescent lesions located in the greater omentum of the same patient as presented in Fig 2, both containing serous adenocarcinoma. All 8 histologically proven malignant metastatic lesions were NIR fluorescent, so detection of metastatic lesions of ovarian cancer with ICG had a sensitivity of 100% in this study (Table 2). The specificity of NIR fluorescence imaging could not be calculated, since lesions that were neither clinically suspect nor fluorescent were not resected.

**Table 2:** Characteristics lesions found with ICG NIR fluorescence imaging.

	Ovarian / tubal carcinomas with metastatic disease N = 2	Ovarian / tubal carcinomas with non-metastatic disease N = 4	Endometrium carcinoma N = 1	Benign N = 3	Total N = 10
**NIR fluorescent lesions**	8	9	2	2	21
**Concordance histopathology**	**N (%)**	**N (%)**	**N (%)**	**N (%)**	**N (%)**
True-positive	8 (100)	0 (0)	0 (0)	0 (0)	8 (38)
False-positive	0 (0)	9 (100)	2 (100)	2 (100)	13 (62)

**Fig 4:**
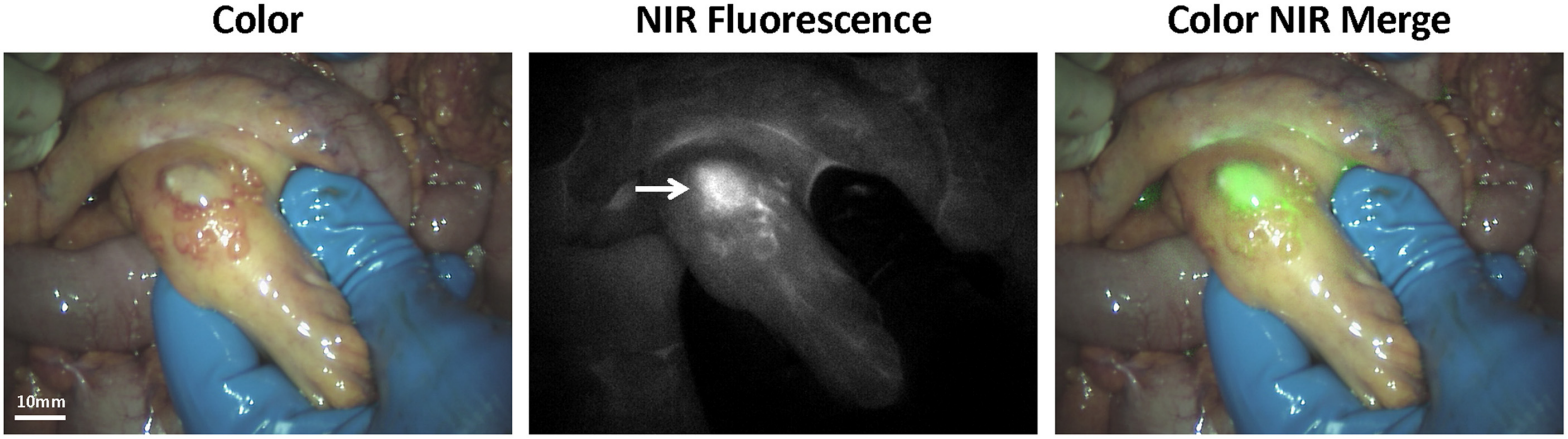
Identification of ovarian cancer omental metastases using NIR fluorescence imaging. A. Identification of a NIR fluorescent lesion located in the mesentery of the intestine. The lesion was classified clinically as a metastasis but was found histologically to be a calcified lymph node.

Clinically none of these 8 malignant and fluorescent lesions had a benign appearance, therefore the use of NIR fluorescence did not lead to the detection of otherwise undetected malignant lesions. In addition 13, on histological assessment, non-malignant lesions were also NIR fluorescent, resulting in a false-positives rate of 62% (Table 2). Of these lesions, 2 were clinically characterized as malignant, 6 as suspicious for malignancy, and 5 as not suspicious for malignancy. Fig 4 shows an example of a NIR fluorescent lesion that was found to be histologically benign, thus a false-positive lesion. This particular lesion was a calcified lymph node. The localization, clinical appearance, pathology, and TBR for each false-positive lesion are listed in Table 3. Globally these false-positive lesions can be divided into two groups: normal tissue (N = 10) and tissue with reactive changes (N = 3).

**Table 3:** Overview of false-positive lesions detected using ICG.

Patient		Localisation	Clinically suspect for malignancy	Pathology	TBR
**Tissue with reactive changes**	2	Mesenterium small bowel	Uncertain	Fibrosis and hemorrhages	2.7
	3	Mesenterium ileum	Yes	Calcified lymph node	2.9*
	6	Ligamentum infundibulum pelvicum left	No	Mature fat and connective tissue, vascular structures, inflammatory infiltrate with giant cell clean-up reaction	2.4
**Healthy tissue**	1	Omentum	Yes	Fat and connective tissue	2.1
	6	Omentum	Uncertain	Muscle	1.3
	8	Peritoneum right	Uncertain	Connective tissue and some tubulair structures	1.6
	8	Iliaca interna right	Uncertain	Lymph node	1.7
	8	Omentum	Uncertain	Muscle	1.6
	9	Bladder peritoneum	No	Fat and connective tissue	1.3
	9	Rectosigmoid	Uncertain	Fat and connective tissue	1.3
	9	Superficial pelvic right	No	Lymph node	1.8
	9	Superficial pelvic left	No	Lymph node	2.6
	9	High paracolic right	No	Fat and connective tissue	†

* Image shown in Fig 4

^†^ TBR could not be calculated

Mean TBR of the fluorescent lesions was 2.0 ± 0.6. There was no significant difference in TBR between histologically confirmed malignant and benign lesions (2.0 vs 2.0; P = 0.99). Although the numbers are small, within the group of false-positive lesions a significant difference in TBR (P = 0.003) did exist between the histologically normal (1.7 ± 0.4) and reactive tissue (2.7 ± 0.2).

No adverse reactions regarding the use of ICG or NIR fluorescence imaging were seen.

## Discussion

In this feasibility study we investigated the use of NIR fluorescence imaging with the clinically available, non-targeted fluorescent tracer ICG in ovarian cancer patients who underwent a surgical staging procedure or cytoreductive surgery. Malignant metastatic lesions were present in 2 out of 10 patients only, but we found that 100% of these histologically proven malignant lesions were fluorescent using this technique. However, there was also a high false-positive rate of 62%.

In cytoreductive surgery, the goal is to remove as much tumor as possible, aiming for complete (no tumor visible after surgery) or leastwise optimal cytoreduction (residual tumor maximized to 10 mm), because the amount of residual tumor is one of the most important prognostic factors for survival in advanced stage patients. Van Dam et al.[14] already showed that with the use of fluorescence imaging using a folate receptor alpha targeting probe (that is over-expressed in 90–95% of ovarian cancer patients), it was possible to identify more tumor deposits than by the naked eye. In our study we could not demonstrate such an added value of NIR fluorescence using the non-specific agent ICG, because all of the fluorescent, histologically malignant lesions were identified with the naked eye.

Treatment decisions in early stage ovarian cancer patients, for instance regarding adjuvant chemotherapy, are based on the presence of occult metastases and the extent of disease found during surgical staging procedures. If staging is not done properly, this could lead to under treatment of the patient. It has been shown that completeness of surgical staging is an independent prognostic factor for overall survival in early stage patients [4]. If the use of NIR fluorescence imaging leads to more accurate detection of (occult) ovarian cancer metastases, more patients could be optimally staged, possibly leading to better treatment decision making and overall survival. In none of the 3 patients with early stage ovarian or fallopian tube cancer who underwent a staging procedure in this study, metastatic disease was found. Therefore, no added value of NIR fluorescence imaging could be demonstrated in this study using ICG. A total of 13 fluorescent lesions were observed in the 7 patients undergoing surgical staging. On pathologic testing, all 13 lesions were benign and thus false-positive. To conclude, NIR fluorescence imaging could not demonstrate added value in staging procedures because no otherwise undetected metastatic disease could be found, but did result in the resection of non-malignant lesions.

The intended effect of NIR fluorescence imaging with ICG was based on the EPR effect. Due to tumor-induced angiogenesis, solid tumors exhibit leaky and immature vessels. Because of this macromolecules are able to permeate through the vessels and into tumor tissue where they are retained due to impaired lymph drainage [16]. ICG is not a macromolecule, but behaves like one after binding to serum proteins. This, combined with the rapid clearance from the circulation, makes ICG a potentially good probe for NIR fluorescence imaging of solid tumors. This theory has proven to be true for gastric and breast cancer [18–23]. But it should be noted that all the trials in breast cancer patients were conducted in a pre-operative diagnostic setting and results may differ from intraoperative usage of ICG.

The large number of false-positives found in this study may be due to the lack of specificity of the EPR effect. It is known that the EPR effect is influenced by multiple factors, such as size, presence of necrosis, tumor type, presence of vascular mediators such as bradykinin or prostaglandins, and location (including primary tumor vs metastatic lesion) of the lesion. Moreover, reactive processes and cancer have parallels in pathophysiological pathways and in vascular mediators. It has been shown that the EPR effect also occurs in inflammatory lesions [25,28,29]. This is in agreement with part of the false-positive lesions identified in the current study. Especially since the EPR effect in reactive tissue is more prominently present hours after injection (coinciding with our imaging window) versus days to weeks after injection in tumor tissue [26].

Several studies describe the intraoperative identification of solid tumors using clinically available, non-targeted fluorescent probes as ICG and methylene blue (MB). These studies report higher TBRs than found in the current study. For example, imaging of colorectal liver metastases using ICG (TBR 7.0) [30], parathyroid adenomas using MB (6.1) [31] and breast cancer using MB (2.4) [32] all showed higher TBRs than the observed 2.0. A possible explanation for this is that in these studies other mechanisms causing accumulation of the fluorescent probe in or around tumor tissue played a role in addition to the possible EPR effect. The EPR effect on its own may not be sufficient in providing high enough TBRs for tumor imaging. Finally, the average TBR of false-positive, benign lesions was just as high as the average TBR of true positive malignant lesions, while the average TBR of reactive benign lesions was even higher than that of malignant lesions. This lack of discriminative power makes NIR fluorescence imaging based on the EPR effect unsuitable for further clinical implementation in ovarian cancer.

## Conclusions

This is the first clinical trial demonstrating the feasibility of intraoperative detection of ovarian cancer metastases using NIR fluorescence imaging and ICG. However, a high number of false-positive lesions that could be explained by the lack of specificity of the EPR effect was found. Moreover, no distinction between malignant, reactive, or benign tissue based on TBR of the different lesions could be made. Therefore, the use of ICG, even when optimized, is not satisfactory for intraoperative NIR fluorescence imaging of ovarian cancer metastases and the need for more tumor-specific targeting agents remains. These results should be confirmed in other solid tumors where intraoperative NIR fluorescence imaging based on the EPR effect is being contemplated.

## Supporting Information

S1 Protocol Study Protocol in Dutch. (DOCX)

S2 Protocol Study Protocol in English. (DOCX)

S1 TREND Checklist TREND Checklist. (PDF)
