# Supplementary material for: The Value of Intraoperative Near-Infrared Fluorescence Imaging Based on Enhanced Permeability and Retention of Indocyanine Green: Feasibility and False-Positives in Ovarian Cancer
Source: PLoS One. 2015 Jun 25;10(6):e0129766. doi: 10.1371/journal.pone.0129766 (PMC4482512; doi:10.1371/journal.pone.0129766)
Supplement: S1 Protocol — (DOCX) [file pone.0129766.s001.docx]

***Addendum P:* Real-time intraoperatieve detectie van metastasen van ovariumcarcinoom met indocyanine groen**

***Parapluprotocol:***

**Real-time intraoperatieve tumordetectie en anatomische mapping met indocyanine groen, methyleen blauw en een nabij-infrarood fluorescentie camerasysteem.**

**25 juli 2012**

**Versie 1.0, Protocolnummer: P10.001**

**Verrichter:**

LUMC, Afdeling Heelkunde, Prof. Dr. C.J.H. van de Velde

**Uitvoerder:**

LUMC, Afdeling Heelkunde, Drs. B.E. Schaafsma

LUMC, Afdeling Heelkunde, Drs. F.P.R. Verbeek

LUMC, Afdeling Heelkunde, Drs. Q.R.J.G. Tummers

**Contactpersonen:**

Dr. A.L. Vahrmeijer [a.l.vahrmeijer@lumc.nl](mailto:a.l.vahrmeijer@lumc.nl) +31 71 526 2309

- Dr. K.N. Gaarenstroom [k.n.gaarenstroom@lumc.nl](mailto:k.n.gaarenstroom@lumc.nl) +31 71 526 1704
- Prof. Dr. A.A.W Peters a.a.w.peters@lumc.nl +31 71 5263348
- Dr. V.T.H.B.M. Smit [v.t.h.b.m.smit@lumc.nl](mailto:v.t.h.b.m.smit@lumc.nl) +31 71 526 6628
- Dr. R.J. Swijnenburg [r.j.swijnenburg@lumc.nl](mailto:r.j.swijnenburg@lumc.nl) +31 71 526 2309
- Drs. J.R. van der Vorst [j.r.van_der_vorst@lumc.nl](mailto:j.r.van_der_vorst@lumc.nl) +31 71 526 4744
  Drs. B.E. Schaafsma [b.e.schaafsma@lumc.nl](mailto:b.e.schaafsma@lumc.nl) +31 71 526 4403
- Drs. F.P.R. Verbeek [f.p.r.verbeek@lumc.nl](mailto:f.p.r.verbeek@lumc.nl) +31 71 526 5401

Drs. Q.R.J.G. Tummers [q.r.j.g.tummers@lumc.nl](mailto:q.r.j.g.tummers@lumc.nl) +31 71 5264744

**Inhoudsopgave**

[1 Samenvatting 4](#_Toc308783904)

[2 Algemeen deel 5](#_Toc308783905)

[2.1 Klinisch probleem 8](#_Toc308783906)

[2.2 Hypothese/vraagstelling 8](#_Toc308783907)

[2.3 Populatie 8](#_Toc308783908)

[2.3.1 Inclusiecriteria 9](#_Toc308783909)

[2.3.2 Exclusiecriteria 9](#_Toc308783910)

[2.3.3 Sample size 9](#_Toc308783915)

[2.4 Studieopzet 9](#_Toc308783916)

[2.5 Indocyanine green dosis 11](#_Toc308783917)

[2.6 Doel 12](#_Toc308783918)

[2.7 Eindpunten 12](#_Toc308783921)

# Samenvatting

**Inleiding**

Stadiering is belangrijk voor het vaststellen van de juiste behandeling en het inschatten van de prognose bij patiënten met ovariumcarcinoom. Het is daarom relevant om occulte metastasen op te sporen in het peritoneum, omentum en retroperitoneale klieren. In het geval van een klinisch laag stadium (I t/m IIa) komen patiënten in aanmerking voor chirurgische behandeling. Indien laag stadium ovariumcarcinoom middels histologisch onderzoek bevestigd wordt kan chemotherapie achterwege worden gelaten.

In geval van een klinisch hoog stadium (IIb t/m IV) is momenteel primaire debulking gevolgd door chemotherapie de standaardbehandeling. In geval van hoog stadium ovariumcarcinoom is het streven om macroscopisch alle metastasen te verwijderen, en indien dit niet lukt te streven naar debulking tot <1cm resttumor in diameter.

Bij het laag stadium ovariumcarcinoom is het van belang om adequaat te stageren en alle microscopische metastasen te detecteren, omdat dit van belang is voor de prognose en behandeling. Hiervoor wordt onder andere gebruikt gemaakt van een stadiëringsoperatie. (www.oncoline.nl/epitheliaal-ovariumcarcinoom)

Recent preklinisch onderzoek heeft laten zien dat het ook mogelijk is om peritoneale metastasen van ovariumcarcinoom zichtbaar te maken met behulp van nabij-infrarode (NIR) fluorescente beeldvorming met indocyanine groen. Wanneer deze resultaten vertaald kunnen worden naar de kliniek zou dit mogelijk kunnen leiden tot betere identificatie van occulte peritoneale, omentale of retroperitoneale metastasen, en daarbij tot een betere pathologische stadiering.

**Vraagstelling**

Kunnen peritoneale, omentale of retroperitoneale ovariumcarcinoom metastasen met het gebruik van NIR fluorescente beeldvorming en indocyanine groen geïdentificeerd worden?

**Populatie**

Binnen deze pilot studie kunnen patiënten worden geïncludeerd met een stadium IIb tot en met IIIc ovariumcarcinoom, waarbij een primaire debulking gepland is. Dit om na te gaan hoe gevoelig de techniek is om peritoneale en omentale metastasen op te sporen. Daarnaast kunnen patiënten geincludeerd worden met een stadium I of IIa ovariumcarcinoom, waarbij een stadiëringsoperatie gepland is.

**Studieopzet**

Patiënten zullen worden verdeeld in two groepen. Een groepen met laag stadium ovariumcarcinoom, en een groepen met hoog stadium ovariumcarcinoom.

Beide groepen van zowel de laag- als hoog stadium zullen tijdens de operatie indocyanine groen toegediend krijgen.

Tijdens de operatie zullen er op verschillende tijdsmomenten opnames worden gemaakt met een NIR fluorescentie camera systeem. Ook na resectie zal het ovariumweefsel op de uitsnijkamer van de pathologie worden bekeken met NIR fluorescente beeldvorming.

**Eindpunten**

Het primair eindpunt is het percentage met NIR fluorescente beeldvorming geïdentificeerde peritoneale, omentale of retroperitoneale ovariumcarcinoom metastasen (het deel van de pathologisch aangetoonde ovarium tumoren dat ook door NIR fluorescente beeldvorming aangetoond wordt)

# Algemeen deel

Ovariumkanker is de 6^e^ meest voorkomende vorm van kanker onder vrouwen. Jaarlijks worden 200.000 vrouwen in de wereld gediagnosticeerd met ovarium kanker. De prognose van deze patiënten is voornamelijk afhankelijk van het stadium (FIGO stadiëring) van de tumor in combinatie met de kwaliteit van de chirurgische behandeling. Patiënten met een laag stadium, FIGO I-IIa, hebben een 5 jaars overleving van 75%-100%. De 5-jaars overleving van patiënten met een hoog stadium, FIGO IIb-IV, bedraagt 20%-60%. (www.oncoline.nl/epitheliaal-ovariumcarcinoom)

Chirurgische resectie en chemotherapie zijn de hoeksteen van de behandeling. Bij optimaal gestadieerde patiënten met stadium I-IIA wordt in de meeste gevallen volstaan met chirurgische resectie van uterus, adnexa en omentum. Er is in Nederland echter geen consensus over het wel of niet geven van chemotherapie in geval er sprake is van graad III tumoren in geval van laag stadium ovariumcarcinoom. Wanneer onvolledig gestadieerd is en restadiering niet mogelijk is, bestaat de behandeling uit combinatie chirurgie en chemotherapie, conform gevorderd stadium ovariumcarcinoom, gezien de aanzienlijke kans op micrometastasen.(www.oncoline.nl/epitheliaal-ovariumcarcinoom)

Echter, ongeveer 70% van de vrouwen presenteren zich met gevorderd stadium ovariumcarcinoom (FIGO IIb-IV). De hoeveelheid tumor die achterblijft na debulking is een van de belangrijkste prognostische factoren in deze patiëntengroep en daarom is het streven naar zo optimaal mogelijke resectie van alle macroscopische tumor. Na resectie krijgen deze patiënten chemotherapie.(1)

Bij chirurgische debulking wordt gestreefd naar macroscopisch geen resttumor, en wanneer dit niet mogelijk is, naar debulking tot <1cm resttumor in diameter. Complete cytoreductie wordt bereikt wanneer er geen tumor meer zichtbaar is na resectie. Indien een complete cytoreductie wordt bereikt, is de overleving aanzienlijk beter in vergelijking met optimale cytoreductie (< 1 cm rest tumor) of suboptimale cytoreductie (> 1 cm resttumor).(2)

Met moderne beeldvormende technieken zoals CT en MRI kunnen tumoren preoperatief redelijk adequaat worden gelokaliseerd en gestadieërd. Met deze beeldvorming en gynaecologisch onderzoek is echter vooraf niet goed een optimale inschatting te maken of er een optimale debulking met achterlaten van < 1 cm tumor rest of tot macroscopisch geen tumor rest kan worden verricht.

In Nederland wordt momenteel onderzocht of het toevoegen van een laparoscopie een beter diagnosticum is om patienten te selecteren die voor primaire debulking in aanmerking komen of voor primaire chemotherapie gevolgd door interval debulking bij gevorderd ovarium carcinoom.(3)

Wanneer sprake is van een klinisch laag ovariumcarcinoom, heeft het opsporen van occulte metastasen grote gevolgen voor de behandeling, aangezien patiënten in geval van metastasen naast chirurgische resectie ook in aanmerking komen voor chemotherapie.(4)

Voor het opsporen van deze metastasen wordt gebruik gemaakt van een stadiëringsoperatie. Deze omvat het verzamelen van biopten van alle plaatsten waarmee de ovariumtumor adhesief of vergroeid is, alle macroscopisch verdachte plaatsen, biopten van het peritoneum en een lymfkliersampling. (www.oncoline.nl/epitheliaal-ovariumcarcinoom)

Een recente ontwikkeling is het gebruik van fluorescente beeldvorming om tumoren ook peroperatief te visualiseren. Deze techniek maakt gebruik van speciale fluorescente kleurstoffen in combinatie met NIR licht. Dit NIR licht is niet zichtbaar voor het menselijk oog, maar kan gedetecteerd worden met een camerasysteem. Het voordeel van deze techniek, is dat er geen gebruikt gemaakt wordt van schadelijke straling, en dat door het gebruik van dit specifieke licht, signaal tot ongeveer 1 cm diep in weefsel gedetecteerd kan worden. Een van de fluorescente kleurstoffen die momenteel goedgekeurd zijn is voor klinisch gebruik is indocyanine groen. Indocyanine groen is een veilige stof en wordt al sinds de jaren vijftig klinisch gebruikt voor onder andere leverfunctie tests en retina angiografie. Daarnaast wordt Indocyanine groen momenteel reeds in onderzoeksverband gebruikt voor NIR fluorescente image-guided surgery voor onder andere detectie van de schildwachtklier bij borst, vulva en lever tumoren.(5)

Omdat indocyanine groen zich in vivo bindt aan serum eiwitten gedraagt het zich als een macromolecuul in de circulatie. Het is bekend dat macromoleculen zich ophopen in tumorweefsel als gevolg van verhoogde vasculaire permeabiliteit en verminderde afvloed. Dit wordt ook wel het “enhanced permeability and retention” (EPR) genoemd.(6;7) In eerdere klinische studies in borstkanker en maagkanker patiënten was het mogelijk om met indocyanine groen in combinatie met optische beeldvorming tumorweefsel te kunnen onderscheiden van het omliggende weefsel.(8-13)

Een recente studie van Kosaka et al.(14) heeft laten zien dat het ook mogelijk is om peritoneale metastasen van ovarium kanker zichtbaar te maken met behulp van NIR fluorescente beeldvorming en intraveneuze injectie van 3,5 mg/kg indocyanine groen in een diermodel. Op basis van deze gegevens zou het mogelijk kunnen zijn om peritoneale, omentale of retroperitoneale metastasen te identificeren met NIR fluorescente beeldvorming na intraveneuze toediening van indocyanine groen.

Daarnaast lieten Ishizawa et al. zien dat het mogelijk was om hepatocellulair carcinoom zichtbaar te maken met behulp van NIR fluorescente beeldvorming en intraveneuze injectie van 0,5 mg/kg indocyanine groen tijdens een laparoscopische hepatectomie.(15)

## Klinisch probleem

Ondanks recente ontwikkelingen op het gebied van preoperatieve beeldvorming blijft het bij intraoperatieve chirurgische stagiering mogelijk om occulte peritoneale, omentale of retroperitoneale metastasen te missen bij vroeg stadium ovariumcarcinoom. Patiënten zouden dan niet adequaat behandeld worden, aangezien in geval van metastasen eigenlijk chemotherapie geïndiceerd is.

Adequate beeldvorming en real-time detectie van deze metastasen bij ovariumcarcinoom kan in potentie de stagering verbeteren. Hierdoor kan mogelijk een betere selectie verricht worden van patiënten die tevens in aanmerking moeten komen voor chemotherapie bij klinisch laag stadium ovariumcarcinoom.

## Hypothese/vraagstelling

Uit preklinisch onderzoek is gebleken dat indocyanine groen gebruikt kan worden voor de identificatie van peritoneale ovariumcarcinoom metastasen. Onze hypothese is dat het om deze reden mogelijk is om ovariumcarcinoom metastasen na intraveneuze injectie met indocyanine groen intraoperatief te kunnen visualiseren met NIR fluorescente beeldvorming.

Vraagstelling:

Is het mogelijk om peritoneale, omentale of retroperitoneale ovariumcarcinoom metastasen te identificeren bij klinisch laag stadium ovariumcarcinoom met NIR fluorescente beeldvorming in combinatie met indocyanine groen?

## Populatie

Binnen deze studie kunnen patiënten worden geïncludeerd met een stadium IIb t/m IIIc ovariumcarcinoom die in aanmerking komen voor primaire cytoreductieve chirurgie. Deze studie dient als pilot, om te onderzoeken hoe haalbaar en betrouwbaar de detectie van metastasen is.

Daarnaast kunnen patiënten geincludeerd worden met een vroeg stadium I-IIa ovariumcarcinoom, om te onderzoeken of het binnen deze patiëntenpopulatie mogelijk is occulte metastasen te identificeren.

### Inclusiecriteria

- Patiënten met verdenking stadium IIb t/m IIIc ovariumcarcinoom die in aanmerking komen voor primaire cytoreductieve chirurgie
- Patiënten met verdenking stadium I of IIa die in aanmerking komen voor een stadiëringsoperatie.
- Leeftijd boven de 18 jaar.

### Exclusiecriteria

# Allergie of overgevoeligheid voor natriumjodide, jodium of ICG

# Patiënten met hyperthyroïdie en patiënten met een autonoom thyroïdaal adenoom

# Patiënten die zwanger zijn

# Patiënten met een ernstige nierinsufficiëntie

### Sample size

In deze fase 2 pilot studie zal de waarde van NIR fluorescente beeldvorming in combinatie met indocyanine groen bij peritoneale, serosale, omentale of retroperitoneale ovarium metastasen worden onderzocht. Omdat deze studie is opgezet als pilot experiment kan geen formele sample size berekening uitgevoerd worden. In totaal zullen er 15 patiënten geïncludeerd worden. Deze zullen worden verdeeld over two groepen. Wij hebben hiervoor gekozen op basis van de heterogeniteit van de ovariumcarcinoom metastasen in de dagelijkse kliniek. Na 15 patiënten verwachten wij een redelijke inschatting te kunnen maken of de detectie van ovariumcarcinoom metastasen met NIR fluorescente beeldvorming en indocyanine groen mogelijk is. Indien wij negatieve resultaten zien bij alle patiënten zullen wij overwegen de pilotstudie voortijdig te stoppen.

## Studieopzet

Alle patiënten gediagnosticeerd met stadium IIb t/m IIIc ovariumcarcinoom die in aanmerking komen voor cytoreductie chirurgie kunnen worden geïncludeerd.

Daarnaast kunnen alle patiënten worden geincludeerd die gediagnosticeerd zijn met stadium I of IIa ovariumcarcinoom en in aanmerking komen voor een stadiëringsoperatie.

Patienten zullen tijdens de operatie indocyanine groen (20 mg) toegediend krijgen (n = 15). Intraoperatieve NIRF beeldvorming zal vergeleken worden met bevindingen van de gynaecoloog tijdens de operatie.

Wanneer door de behandelend gynaecoloog besloten wordt ook laparoscopische stadiering toe te passen (al dan niet in het kader van de LAPOVCA studie), zal hier tevens laparoscopische intraoperatieve NIRF beeldvorming voor gebruikt worden. (Zie parapluprotocol P10.001, versie 3.0, appendix 4 en 5 voor een uitgebreide beschrijving van het laparoscopisch camerasysteem).

Aanvullende NIR fluorescente spots niet opgemerkt bij macroscopie zullen tevens verwijderd worden en opgestuurd worden voor pathologie. Het preparaat zal direct na de resectie vervoerd worden naar de afdeling pathologie van het LUMC. Hier zullen de resectie preparaten nogmaals macroscopische gefotografeerd worden en direct opnieuw met NIR fluorescente imaging bekeken worden. Vervolgens zal de normale procedure ter beoordeling van het resectiepreparaat geldende bij de afdeling pathologie gevolgd worden, ook de macroscopisch niet opgemerkte NIR positieve spots zullen pathologisch onderzocht worden.

**Stadium IIb t/m III ovariumcarcinoom waarbij een debulking zal worden uitgevoerd**

**Stadium I en IIa ovariumcarcinoom waarbij een stadiëringsoperatie zal worden uitgevoerd**

**In-/exclusie criteria**

**I.V. injectie Indocyanine green**

**NIR imaging**

**Resectie door gynaecoloog**

**Resectiepreparaat naar uitsnijkamer pathologie**

**NIR imaging**

**Fixeren**

*Figuur 1. Schematische weergave studieopzet*

## Indocyanine green dosis

De dosis die gebruikt zal worden is 20 mg indocyanine groen. Deze hoeveelheid indocyanine groen zal net voor het toedienen opgelost worden in water. De indocyanine groen zal door een van de arts-onderzoekers intraveneus toegediend worden. De indocyanine groen zal peroperatief toegediend worden. De hoeveelheid ICG blijft daarbij ruim onder de maximale dagelijkse dosering ICG van 5mg/kg (bijsluiter ICG-Pulsion). Toediening zal gebeuren in een bolus. De dosering is gebaseerd op resultaten die zijn behaald in preklinische modellen waar gebruik werd gemaakt van 3.5 mg/kg en het feit dat boven de 0.5 mg/kg het aantal allergische reacties op indocyanine groen toenemen.(16) In eerdere studies met tumor imaging middels indocyanine groen en optische beeldvorming werd 0.01 – 0.25 mg/kg indocyanine groen gebruikt.(9;10) Echter hier werd alleen gekeken tot op 30 min na injectie.

## Doel

### Primair Doel

Het primair doel van deze studie is het beter identificeren van peritoneale, serosale, omentale of retroperitoneale metastasen bij ovariumcarcinoom met indocyanine groen.

### Secundair doel

- Bepalen of het mogelijk om extra peritoneale, omentale of retroperitoneale ovariumcarcinoom metastasen te detecteren middels NIR fluorescente beeldvorming.

## Eindpunten

**Primair eindpunt**

Het primair eindpunt is het percentage geïdentificeerde metastasen ovariumcarcinoom (het deel van de pathologisch aangetoonde tumoren dat ook door NIRF imaging aangetoond wordt).

### Secundaire eindpunten

- Tumor-to-background ratio tussen gezond en tumorweefsel.
- Aantal nieuw gedetecteerde laesies met behulp van NIR fluorescente beeldvorming

**Reference list**

(1) Elattar A, Bryant A, Winter-Roach BA, Hatem M, Naik R. Optimal primary surgical treatment for advanced epithelial ovarian cancer

Cochrane Database Syst Rev 2011;(8):CD007565.

(2) Bristow RE, Chi DS. Platinum-based neoadjuvant chemotherapy and interval surgical cytoreduction for advanced ovarian cancer: a meta-analysis. Gynecol Oncol 2006 Dec;103(3):1070-6.

(3) Rutten MJ, Gaarenstroom KN, Van Gorp T, van Meurs HS, Arts HJ, Bossuyt PM, et al. Laparoscopy to predict the result of primary cytoreductive surgery in advanced ovarian cancer patients (LapOvCa-trial): a multicentre randomized controlled study. BMC Cancer 2012;12:31.

(4) Trimbos JB, Vergote I, Bolis G, Vermorken JB, Mangioni C, Madronal C, et al. Impact of adjuvant chemotherapy and surgical staging in early-stage ovarian carcinoma: European Organisation for Research and Treatment of Cancer-Adjuvant ChemoTherapy in Ovarian Neoplasm trial. J Natl Cancer Inst 2003 Jan 15;95(2):113-25.

(5) Schaafsma BE, Mieog JSD, Hutteman M, van der Vorst JR, Kuppen PJ, Lowik CW, et al. The clinical use of indocyanine green as a near-infrared fluorescent contrast agent for image-guided oncologic surgery. J Surg Oncol 2011;in press.

(6) Matsumura Y, Maeda H. A new concept for macromolecular therapeutics in cancer chemotherapy: mechanism of tumoritropic accumulation of proteins and the antitumor agent smancs. Cancer Res 1986 Dec;46(12 Pt 1):6387-92.

(7) Maeda H, Wu J, Sawa T, Matsumura Y, Hori K. Tumor vascular permeability and the EPR effect in macromolecular therapeutics: a review. J Control Release 2000 Mar 1;65(1-2):271-84.

(8) Hagen A, Grosenick D, Macdonald R, Rinneberg H, Burock S, Warnick P, et al. Late-fluorescence mammography assesses tumor capillary permeability and differentiates malignant from benign lesions. Opt Express 2009 Sep 14;17(19):17016-33.

(9) Poellinger A, Burock S, Grosenick D, Hagen A, Ludemann L, Diekmann F, et al. Breast cancer: early- and late-fluorescence near-infrared imaging with indocyanine green--a preliminary study

Radiology 2011 Feb;258(2):409-16.

(10) Kimura T, Muguruma N, Ito S, Okamura S, Imoto Y, Miyamoto H, et al. Infrared fluorescence endoscopy for the diagnosis of superficial gastric tumors

Gastrointest Endosc 2007 Jul;66(1):37-43.

(11) Ntziachristos V, Yodh AG, Schnall M, Chance B. Concurrent MRI and diffuse optical tomography of breast after indocyanine green enhancement. Proc Natl Acad Sci U S A 2000 Mar 14;97(6):2767-72.

(12) Alacam B, Yazici B, Intes X, Nioka S, Chance B. Pharmacokinetic-rate images of indocyanine green for breast tumors using near-infrared optical methods. Phys Med Biol 2008 Feb 21;53(4):837-59.

(13) Mataki N, Nagao S, Kawaguchi A, Matsuzaki K, Miyazaki J, Kitagawa Y, et al. Clinical usefulness of a new infrared videoendoscope system for diagnosis of early stage gastric cancer

Gastrointest Endosc 2003 Mar;57(3):336-42.

(14) Kosaka N, Mitsunaga M, Longmire MR, Choyke PL, Kobayashi H. Near infrared fluorescence-guided real-time endoscopic detection of peritoneal ovarian cancer nodules using intravenously injected indocyanine green

Int J Cancer 2011 Oct 1;129(7):1671-7.

(15) Ishizawa T, Bandai Y, Harada N, Muraoka A, Ijichi M, Kusaka K, et al. Indocyanine green-fluorescent imaging of hepatocellular carcinoma during laparoscopic hepatectomy: An initial experience. Asian Journal of Endoscopic Surgery 2010;3(1):42-5.

(16) Speich R, Saesseli B, Hoffmann U, Neftel KA, Reichen J. Anaphylactoid reactions after indocyanine-green administration

Ann Intern Med 1988 Aug 15;109(4):345-6.
