# Supplementary material for: The Value of Intraoperative Near-Infrared Fluorescence Imaging Based on Enhanced Permeability and Retention of Indocyanine Green: Feasibility and False-Positives in Ovarian Cancer
Source: PLoS One. 2015 Jun 25;10(6):e0129766. doi: 10.1371/journal.pone.0129766 (PMC4482512; doi:10.1371/journal.pone.0129766)
Supplement: S2 Protocol — (DOCX) [file pone.0129766.s002.docx]

***Addendum P:* Real-time intraoperative detection of ovarian cancer metastases using indocyanine green**

***Umbrella protocol:***

**Real-time intraoperative tumor detection and anatomical mapping using indocyanine green, methylene blue and a near-infrared fluorescence imaging system.**

**25 July 2012**

**Version 1.0, Protocol number: P10.001**

**Provider:**

LUMC, Afdeling Heelkunde, Prof. Dr. C.J.H. van de Velde

**Executor:**

LUMC, Dept. Surgery, Drs. B.E. Schaafsma

LUMC, Dept. Surgery, Drs. F.P.R. Verbeek

LUMC, Dept. Surgery, Drs. Q.R.J.G. Tummers

**Contact persons:**

Dr. A.L. Vahrmeijer [a.l.vahrmeijer@lumc.nl](mailto:a.l.vahrmeijer@lumc.nl) +31 71 526 2309

- Dr. K.N. Gaarenstroom [k.n.gaarenstroom@lumc.nl](mailto:k.n.gaarenstroom@lumc.nl) +31 71 526 1704
- Prof. Dr. A.A.W Peters a.a.w.peters@lumc.nl +31 71 5263348
- Dr. V.T.H.B.M. Smit [v.t.h.b.m.smit@lumc.nl](mailto:v.t.h.b.m.smit@lumc.nl) +31 71 526 6628
- Dr. R.J. Swijnenburg [r.j.swijnenburg@lumc.nl](mailto:r.j.swijnenburg@lumc.nl) +31 71 526 2309
- Drs. J.R. van der Vorst [j.r.van_der_vorst@lumc.nl](mailto:j.r.van_der_vorst@lumc.nl) +31 71 526 4744
  Drs. B.E. Schaafsma [b.e.schaafsma@lumc.nl](mailto:b.e.schaafsma@lumc.nl) +31 71 526 4403
- Drs. F.P.R. Verbeek [f.p.r.verbeek@lumc.nl](mailto:f.p.r.verbeek@lumc.nl) +31 71 526 5401

Drs. Q.R.J.G. Tummers [q.r.j.g.tummers@lumc.nl](mailto:q.r.j.g.tummers@lumc.nl) +31 71 5264744

**Content**

[1 Summary 4](#_Toc308783904)

[2 Introduction 5](#_Toc308783905)

[2.1 Clinical problem 8](#_Toc308783906)

[2.2 Hypothesis/Aim 8](#_Toc308783907)

[2.3 Population 8](#_Toc308783908)

[2.3.1 Inclusioncriteria 9](#_Toc308783909)

[2.3.2 Exclusioncriteria 9](#_Toc308783910)

[2.3.3 Sample size 9](#_Toc308783915)

[2.4 Trial design 9](#_Toc308783916)

[2.5 Indocyanine green dosis 11](#_Toc308783917)

[2.6 Objective 12](#_Toc308783918)

[2.7 Endpoints 12](#_Toc308783921)

# Summary

**Introduction**

Staging is important for determining the appropriate treatment and for estimating the prognosis in patients with ovarian cancer. It is therefore relevant to detect occult metastasis in the peritoneum, omentum, and retroperitoneal nodes. In case of low stage ovarian cancer (I t / m IIa) patients are eligible for surgical treatment. If low-stage ovarian cancer is confirmed by histological examination, chemotherapy can be omitted.

In case of high stage ovarian cancer (IIb t / m IV) primary debulking is standardly followed by chemotherapy. In case of high stage ovarian cancer, the aim is to remove all macroscopic metastases. And, if this not possible, to strive for debulking to <1 cm residual tumor diameter.

For low stage ovarian cancer, it is important to perform an accurate staging procedure to detect all microscopic metastases, as this is of importance for the prognosis and further treatment. Hence a surgical staging procedure is performed. (www.oncoline.nl/epitheliaal-ovariumcarcinoom)

Recent preclinical studies have shown that it is possible to detect peritoneal ovarian cancer metastases using near-infrared (NIR) fluorescence imaging with indocyanine green (ICG). If these results can be translated to the clinic, this could possibly lead to better identification of occult peritoneal, omental and retroperitoneal metastases, and consequently to better staging.

**Research Question**

Is it possible to identify occult peritoneal, omental and retroperitoneal ovarian cancer metastases by NIR fluorescence imaging using indocyanine green?

**Population**

In order to verify the sensitivity of the technique in detecting omental and peritoneal metastases patients with stage IIb to IIIc ovarian cancer planned for a debulking procedure will be enrolled in this pilot study. In addition, patients with stage I or IIa ovarian cancer planned for a surgical staging procedure will be enrolled.

**Trial design**

Patients will be divided into two groups. One group with low stage ovarian cancer, and one group with high stage ovarian cancer. All patients in both groups will be administered ICG during at start of the surgery.

During surgery imaging with a NIR fluorescence camera system will be performed at different time points. After resection, NIR fluorescence imaging of the resected tissue will be performed at the pathology department.

**End points**

The primary endpoint is the percentage of peritoneal, omental or retroperitoneal ovarian cancer metastases identified with NIR fluorescence imaging (defined as the part of the pathology proven ovarian cancer metastases that also show NIR fluorescence)

# Introduction

Ovarian cancer is the 6th most common cancer among women. Worldwide every year 200,000 women are diagnosed with ovarian cancer. The prognosis of these patients is primarily dependent on the stage (FIGO staging) of the tumor, and of the accuracy of the surgical treatment. Patients with low stage ovarian cancer (FIGO I-IIa) have a 5-year survival of 75% -100%. The 5-year survival of patients with high stage ovarian cancer (FIGO IIb-IV) is 20% -60%. ([www.oncoline.nl/epitheliaal-ovariumcarcinoom](http://www.oncoline.nl/epitheliaal-ovariumcarcinoom))

Surgical resection and chemotherapy are the cornerstones of the treatment of ovarian cancer. For low stage ovarian cancer surgical resection of the uterus, adnexa and omentum is sufficient in most cases, provided that the surgical staging is optimally performed. In the Netherlands, no consensus exists on whether to give chemotherapy in case of low stage ovarian cancer with a grade III tumor. When staging is incomplete and restaging is not possible, low stage ovarian cancer is treated like high stage ovarian cancer with surgery followed by chemotherapy, due to the significant risk of micro metastases. (www.oncoline.nl/epitheliaal-ovariumcarcinoom)

However, about 70% of women present with advanced stage ovarian cancer (FIGO IIb-IV). The amount of tumor remaining after debulking is the most important prognostic factors in this group of patients and therefore the goal of surgery is to completely resect all tumor. After resection, these patients receive chemotherapy.(1)

In surgical debulking the aim is to leave no macroscopic residual tumor, and when this is not possible, to only leave residual tumor <1 cm in diameter. Complete surgical debulking is achieved when no tumor is visible after resection. After a complete debulking surgery, the survival rate is significantly better in comparison to optimal (<1 cm residual tumor), or sub-optimal (> 1 cm residual tumor) debulking surgery.(2)

With modern imaging techniques, such as CT and MRI, preoperative localization and staging of tumors can be done reasonably well. However, preoperative imaging and gynecological examination alone are not sufficient in estimating whether a complete or optimal debulking can be carried out. Therefor in the Netherlands, it is currently investigated whether a preoperative laparoscopy can contribute to a better distinction between patients eligible for primary debulking and patients eligible for neo-adjuvant chemotherapy followed by interval debulking in advanced ovarian cancer.(3)

In patients with low stage ovarian cancer, detecting occult metastases has major implications for the treatment, as treatment of patients with metastasised disease involves chemotherapy after the surgical resection.(4)

In order to detect these metastases a staging surgery is performed. This involves taking biopsies of any adhesions surrounding the (area of the) primary tumour, all macroscopic suspect areas, blind biopsies of amongst others the peritoneum, and lymph node sampling. (www.oncoline.nl/epitheliaal-ovariumcarcinoom)

A recent development is the use of fluorescence imaging to visualize also tumors during surgery. This technique makes use of special fluorescent dyes, in combination with NIR light. This NIR light is not visible to the human eye, but can be detected with a camera system. The advantages of this technique are that it does not involve the use of harmful radiation, yet has a penetration depth of approximately 1 cm in tissue. One of the fluorescent dyes which is currently approved for clinical use is ICG. ICG is considered safe for use in humans and has been clinically used since the nineteen fifties for liver function tests and retinal angiography. Moreover, ICG has already been used in image-guided surgery research into detection of the sentinel node in breast, vulva and liver tumors with NIR fluorescence.(5)

Because in vivo ICG binds to serum proteins, it behaves as a macromolecule in the circulation. It is known that macromolecules accumulate in tumor tissue due to increased vascular permeability and reduced drainage in these tissues. This is known as the "enhanced permeability and retention" (EPR) effect.(6;7) In previous clinical trials in breast and gastric cancer patients it was possible to distinguish tumor tissue from the surrounding tissue using ICG in combination with optical imaging.(8-13)

A recent study of Kosaka et al.(14) has shown that in an animal model it is possible to detect peritoneal metastases of ovarian cancer with NIR fluorescent imaging after an intravenous injection of 3.5 mg / kg of ICG. Based on the above mentioned data, it may also be possible to identify peritoneal, omental or retroperitoneal metastases in ovarian cancer patients with NIR fluorescent imaging after intravenous administration of ICG.

In addition, Ishizawa et al. demonstrated successful visualization of hepatocellular carcinoma using NIR fluorescent imaging and intravenous injection of 0.5 mg/kg ICG during a laparoscopic hepatectomy.(15)

## Clinical Problem

Despite recent advancements in preoperative imaging, it remains challenging to detect peritoneal, omental and retroperitoneal metastases during staging surgery for early stage ovarian cancer. Undetected metastasized disease could lead to inadequate treatment of patients, due to the unjustified of chemotherapy.

Adequate imaging and real-time detection of these metastases could possibly improve the surgical staging procedure, consequently permitting a better selection of patients that should receive additional chemotherapy.

## Hypothesis / aim

Preclinical studies have shown that ICG can be used for the identification of peritoneal ovarian carcinoma metastases. Therefor we have the hypothesis that in after intravenous injection of ICG it is possible to intraoperatively detect ovarian cancer metastases using NIR fluorescent imaging.

Research Question:

Is it possible to identify peritoneal, omental or retroperitoneal ovarian cancer metastases in clinical low stage ovarian cancer patients using NIR fluorescence imaging and ICG?

## Population

In this study, patients with stage IIb t / m IIIc ovarian cancer who are eligible for primary debulking surgery will be enrolled to study feasibility of detection of metastases. In addition, patients with early stage I-IIa ovarian cancer will be enrolled to investigate if identification of occult metastases is possible within this patient population.

.

### Inclusion criteria

- Patients with suspected stage IIb t / m IIIc ovarian cancer who are eligible for primary debulikng surgery
- Patients with suspected stage I or IIa eligible for a surgical staging procedure.
- Age over 18 years.

### Exclusion criteria

- Allergy or hypersensitivity to sodium iodide, iodine or ICG
- Hyperthyroidism and autonomous thyroid adenoma
- Pregnancy
- Severe renal impairment

### Sample size

In this phase 2 pilot study, the value of NIR fluorescent imaging in detection of peritoneal, omental and retroperitoneal ovarian metastases after ICG injection will be studied. Because this study was designed as a pilot study no formal sample size calculation could be performed. Based on the heterogeneity of ovarian cancer metastases in the daily clinic, we expect a total of 15 patients will need to be enrolled. With this sample size we should be able to make a reasonable estimate of the feasibility of the detection of ovarian cancer metastases with NIR fluorescence imaging and ICG. If negative results are seen in all patients, we will consider stopping the pilot study prematurely.

## Study design

All patients diagnosed with stage IIb t / m IIIc ovarian cancer eligible for debulking surgery will be included. In addition, all patients diagnosed with stage I or IIa ovarian cancer eligble for a surgical staging procedure will be enrolled.

All patients (n = 15) will be administered ICG (20 mg) during the surgery. Intraoperative NIR fluorescence imaging findings will be compared with clinical findings by the gynecologist during the surgery.

During the surgery, standard procedure will be followed and biopsies of clinically suspected areas will be obtained. Additional NIR fluorescent spots, that were not detected by the gynaecologist with usual visual/tactile methods, will also be removed and sent to the pathology department for histopathological assessment. Immediately after the resection, the resected tissue specimens will be transported to the pathology department of the LUMC, where specimens will be photographed and additional NIR fluorescent imaging will be performed. Hereafter the department of pathology’s standard procedure for assessing the resected specimens will be followed for both specimens detected under usual methods and specimens detected under NIR fluorescence imaging.

When it is decided by the attending gynaecologist to also perform a laparoscopic staging (whether or not in the context of the LAPOVCA study), a laparoscopic intra-operative NIR fluorescence imaging system can be used. (See umbrella protocol P10.001 protocol, version 3.0, Appendix 4 and 5 for a detailed description of the laparoscopic camera system).

**Stage IIb to III ovarian cancer scheduled for debulking surgery**

**Stage I and IIa ovarian cancer scheduled for surgical staging procedure**

**In-/exclusion criteria**

**I.V. injection Indocyanine green**

**NIR imaging**

**Resection by gynaecologist**

**Resection specimen to pathology department**

**NIR imaging**

**Histopathological assesment**

*Figure 1. Schematic overview study design*

## Indocyanine green dose

The dose to be used in this study is 20 mg indocyanine green. This amount of indocyanine green will be dissolved in water just prior to administration. The indocyanine green will be administered intravenously by the anesthesiologist during the surgery. The amount of ICG remains well below the maximum daily dose of ICG 5mg / kg (ICG-Pulsion leaflet). Administration will be done in a single bolus. The dose is based on results of preclinical models in which a dose of 3.5 mg / kg was used, keeping in mind that with doses above the 0.5 mg / kg, the number of allergic reactions to ICG increase.(16)

In previous studies regarding optical imaging of tumor tissue with ICG a dose of 0.01 - 0.25 mg/kg ICG was used.(9;10) However, in these studies NIR fluorescence imaging was performed 30 min after injection.

## Objectives

### Primary Objective

The primary goal of this study is to improve the intra-operative detection of peritoneal, omental and retroperitoneal metastases in ovarian cancer patients using ICG.

### Secondary Objective

The secondary goal is to determine whether additional peritoneal, omental, retroperitoneal ovarian cancer metastases can be detected with NIR fluorescent imaging after ICG injection.

## End points

**Primary End Point**

The primary endpoint is the percentage of peritoneal, omental or retroperitoneal ovarian cancer metastases identified with NIR fluorescence imaging (defined as the part of the pathology proven ovarian cancer metastases that also show NIR fluorescence)

### Secondary End Points

- Tumor-to-background ratio between tumor and healthy tissue.
- Number of additional additional peritoneal, omental, retroperitoneal ovarian cancer metastases detected with NIR fluorescent imaging after ICG injection.

**Reference list**

(1) Elattar A, Bryant A, Winter-Roach BA, Hatem M, Naik R. Optimal primary surgical treatment for advanced epithelial ovarian cancer

Cochrane Database Syst Rev 2011;(8):CD007565.

(2) Bristow RE, Chi DS. Platinum-based neoadjuvant chemotherapy and interval surgical cytoreduction for advanced ovarian cancer: a meta-analysis. Gynecol Oncol 2006 Dec;103(3):1070-6.

(3) Rutten MJ, Gaarenstroom KN, Van Gorp T, van Meurs HS, Arts HJ, Bossuyt PM, et al. Laparoscopy to predict the result of primary cytoreductive surgery in advanced ovarian cancer patients (LapOvCa-trial): a multicentre randomized controlled study. BMC Cancer 2012;12:31.

(4) Trimbos JB, Vergote I, Bolis G, Vermorken JB, Mangioni C, Madronal C, et al. Impact of adjuvant chemotherapy and surgical staging in early-stage ovarian carcinoma: European Organisation for Research and Treatment of Cancer-Adjuvant ChemoTherapy in Ovarian Neoplasm trial. J Natl Cancer Inst 2003 Jan 15;95(2):113-25.

(5) Schaafsma BE, Mieog JSD, Hutteman M, van der Vorst JR, Kuppen PJ, Lowik CW, et al. The clinical use of indocyanine green as a near-infrared fluorescent contrast agent for image-guided oncologic surgery. J Surg Oncol 2011;in press.

(6) Matsumura Y, Maeda H. A new concept for macromolecular therapeutics in cancer chemotherapy: mechanism of tumoritropic accumulation of proteins and the antitumor agent smancs. Cancer Res 1986 Dec;46(12 Pt 1):6387-92.

(7) Maeda H, Wu J, Sawa T, Matsumura Y, Hori K. Tumor vascular permeability and the EPR effect in macromolecular therapeutics: a review. J Control Release 2000 Mar 1;65(1-2):271-84.

(8) Hagen A, Grosenick D, Macdonald R, Rinneberg H, Burock S, Warnick P, et al. Late-fluorescence mammography assesses tumor capillary permeability and differentiates malignant from benign lesions. Opt Express 2009 Sep 14;17(19):17016-33.

(9) Poellinger A, Burock S, Grosenick D, Hagen A, Ludemann L, Diekmann F, et al. Breast cancer: early- and late-fluorescence near-infrared imaging with indocyanine green--a preliminary study

Radiology 2011 Feb;258(2):409-16.

(10) Kimura T, Muguruma N, Ito S, Okamura S, Imoto Y, Miyamoto H, et al. Infrared fluorescence endoscopy for the diagnosis of superficial gastric tumors

Gastrointest Endosc 2007 Jul;66(1):37-43.

(11) Ntziachristos V, Yodh AG, Schnall M, Chance B. Concurrent MRI and diffuse optical tomography of breast after indocyanine green enhancement. Proc Natl Acad Sci U S A 2000 Mar 14;97(6):2767-72.

(12) Alacam B, Yazici B, Intes X, Nioka S, Chance B. Pharmacokinetic-rate images of indocyanine green for breast tumors using near-infrared optical methods. Phys Med Biol 2008 Feb 21;53(4):837-59.

(13) Mataki N, Nagao S, Kawaguchi A, Matsuzaki K, Miyazaki J, Kitagawa Y, et al. Clinical usefulness of a new infrared videoendoscope system for diagnosis of early stage gastric cancer

Gastrointest Endosc 2003 Mar;57(3):336-42.

(14) Kosaka N, Mitsunaga M, Longmire MR, Choyke PL, Kobayashi H. Near infrared fluorescence-guided real-time endoscopic detection of peritoneal ovarian cancer nodules using intravenously injected indocyanine green

Int J Cancer 2011 Oct 1;129(7):1671-7.

(15) Ishizawa T, Bandai Y, Harada N, Muraoka A, Ijichi M, Kusaka K, et al. Indocyanine green-fluorescent imaging of hepatocellular carcinoma during laparoscopic hepatectomy: An initial experience. Asian Journal of Endoscopic Surgery 2010;3(1):42-5.

(16) Speich R, Saesseli B, Hoffmann U, Neftel KA, Reichen J. Anaphylactoid reactions after indocyanine-green administration

Ann Intern Med 1988 Aug 15;109(4):345-6.
